# Heat Health Management in a Quarantine and Isolation Facility in the Tropics

**DOI:** 10.1017/S1049023X22000255

**Published:** 2022-04

**Authors:** Dianne Stephens, Matt Brearley, Lisa Vermeulen

**Affiliations:** 1. National Critical Care and Trauma Response Centre, Darwin, Northern Territory, Australia; 2. Northern Territory Department of Health, Centre for National Resilience, Casuarina, Northern Territory, Australia

**Keywords:** COVID-19, emergency workers, heat health, quarantine facility, tropics

## Abstract

**Introduction:**

The Howard Springs Quarantine Facility (HSQF) is located in tropical Northern Australia and has 875 blocks of four rooms (3,500 rooms in total) spread over 67 hectares. The HSQF requires a large outdoor workforce walking outdoor pathways to provide individual care in the ambient climate. The personal protective equipment (PPE) required for the safety of quarantine workers varies between workgroups and limits body heat dissipation that anecdotally contributes to excessive sweating, which combined with heat stress symptoms of fatigue, headache, and irritability, likely increases the risk of workplace injuries including infection control breaches.

**Study Objective:**

The purpose of this study was the description of qualitative and quantitative assessment for HSQF workers exposed to tropical environmental conditions and provision of evidenced-based strategies to mitigate the risk of heat stress in an outdoor quarantine and isolation workforce.

**Methods:**

The study comprised two components - a cross-sectional physiological monitoring study of 18 workers (eight males/ten females; means: 41.4 years; 1.69m; 80.6kg) during a single shift in November 2020 and a subjective heat health survey completed by participants on a minimum of four occasions across the wet season/summer period from November 2020 through February 2021. The physiological monitoring included continuous core temperature monitoring and assessment of fluid balance.

**Results:**

The mean apparent temperature across first-half and second-half of the shift was 34.7°C (SD = 0.8) and 35.6°C (SD = 1.9), respectively. Across the work shift (mean duration 10.1 hours), the mean core temperature of participants was 37.3°C (SD = 0.2) with a range of 37.0°C - 37.7°C. The mean maximal core temperature of participants was 37.7°C (SD = 0.3). In the survey, for the workforce in full PPE, 57% reported feeling moderately, severely, or unbearably hot compared to 49% of those in non-contact PPE, and the level of fatigue was reported as moderate to severe in just over 25% of the workforce in both groups.

**Conclusion:**

Heat stress is a significant risk in outdoor workers in the tropics and is amplified in the coronavirus disease 2019 (COVID-19) frontline workforce required to wear PPE in outdoor settings. A heat health program aimed at mitigating risk, including workplace education, limiting exposure times, encouraging hydration, buddy system, active cooling, and monitoring, is recommended to limit PPE breaches and other workplace injuries in this workforce.

## Introduction

Darwin is the capital city of the Northern Territory (NT) of Australia and is located in the far north of Australia. Darwin hosts the National Critical Care and Trauma Response Centre (NCCTRC) which equips, manages, and coordinates the Australian Government’s health emergency response capability Australian Medical Assistance Teams (AUSMATs). In February 2020 at the very outset of the coronavirus disease 2019 (COVID-19) pandemic, the Australian Government required a quarantine destination for repatriated Australians caught in the COVID-19 outbreak epicenter in Wuhan, China. A residential workers’ village located 30km outside Darwin city in Howard Springs was repurposed to house these evacuees. The Howard Springs Quarantine Facility (HSQF) has 875 blocks of four rooms sharing a common balcony spread over 67 hectares – there are a total of 3,500 rooms. The rooms are arranged into sectors, each with a large communal laundry facility. In response to the transmission events occurring in commercial hotel quarantine facilities, the NT Chief Health Officer directed from July 2020 that all people entering the NT from a declared COVID-19 hotspot would undertake mandatory supervised quarantine for 14 days in one of two facilities – the HSQF in Darwin or the Todd Quarantine Facility in Alice Springs (NT, Australia).

The HSQF offers improved infection control and more effective prevention of transmission events when compared to commercial hotel accommodations with reduced touch points, no lifts or stair wells, verandas outside every room, independent air conditioning, and wide outdoor corridors distanced from room doors for transit of quarantine workforce through the zones.^1,2^ The unique challenges of providing a quarantine and isolation facility over a large site in the tropics included implementation of infection prevention hierarchy of controls in a predominantly outdoor work environment, long distances to be covered by workers within the quarantine zones, environmental health hazards such as biting insects and poisonous snakes, exposure to the seasonal hazards such as monsoonal rains, violent storms, and cyclones in the wet season, and the constant exposure throughout the year to heat and humidity.

The quarantine and isolation workforce includes health, welfare, police, and several private contractor groups including security, catering, cleaning, concierge, maintenance, and tradespeople. The quarantine facility is divided into zones related to the transmission potential of the work within that zone with an infection prevention hierarchy of controls applied to mitigate risk specific to each zone. The Red Zone accommodates the well COVID-19 positive people who do not require hospital care and their close contacts as defined by Communicable Diseases Network Australia Series of National Guidelines (CDNA SoNG).^3^ The Orange Zones accommodate all people in quarantine and are separated into 12 areas to provide cohorting of different groups of arrivals. The Green Zone is any area that is not Red or Orange and includes entry and exit points to Red and Orange Zones, administration areas, catering preparation areas, and staff accommodation. Infection control measures enforced across all the zones on the site include regular staff refresher training in personal protective equipment (PPE), stay home and get tested orders for anyone unwell, daily polymerase chain reaction (PCR) self-testing, strict hand hygiene, physical distancing, regular sanitizing of surfaces, shared resources and common touch points, single use consumables, and individual serves for staff catering. The Red and Orange Zones have additional PPE requirements with donning and doffing stations located outside each defined zone. Staff entering the Red Zone or providing health care in the Orange Zone must donn long sleeved impervious gown, P2/N95 mask (type of half-face particulate respirators), eye protection (goggles or face shield), and long cuff gloves. Staff entering the Orange Zones but remaining physically distanced from residents at all times donn P2/N95 mask, eye protection (goggles or face shield), and gloves.

The COVID-19 pandemic has generated a need for many medical clinics to set up triage and treatment facilities outdoors, which in hot climates has resulted in heat stress in workers and febrile patients.^4^ The HSQF workforce spend most of their shift outside in the ambient heat and humidity in PPE. The impervious gowns trap heat, masks become ineffective if damp, and gloves reduce manual dexterity and trap water from sweaty hands – a combination of factors that increase the risk of a PPE breach and a transmission event occurring amongst the workforce. Furthermore, exposure to the tropical environment for outdoor workers can result in heat-related symptoms that include fatigue, headache, and irritability that have been termed a “heat hangover.”^5,6^ While the workplace consequences of such symptoms are yet to be thoroughly determined, they are likely related to the rise in workplace injuries during hot weather.^7^

The objectives of the study are to describe the impact of ambient heat and humidity on the quarantine workforce in the tropics, to outline methods for quantifying heat stress in the workforce, and to provide strategies to mitigate the risk of heat stress in an outdoor quarantine and isolation workforce.

## Methods

### Ethical Review

The study was reviewed by the Human Research Ethics Committee of the NT Department of Health (Darwin City, NT, Australia) and Menzies School of Health Research (HREC; Casuarina, NT, Australia) in accordance with the National Health and Medical Research Council (NHMRC; Canberra, Australian Capital Territory) National Statement on Ethical Conduct in Human Research 2007 and was granted full ethical approval on November 10, 2020 (HREC 2020-3897).

### Field Study Design

The field study comprised two components - a cross-sectional physiological monitoring of 18 workers on a single shift in November 2020 and a subjective heat health survey completed by participants on a minimum of four occasions across the November 2020 through February 2021 period. All workers on site were provided with information about the study through heat health information sessions and in-house social media posts. All workers were invited to participate anonymously in the survey through a QR-code application-based program and volunteers were sought through information sessions and flyers posted around the site for the physiological monitoring component of the study. All outdoor workers available on the day of physiological testing were eligible for inclusion in the study.

### Physiological and Perceptual

Gastrointestinal temperature (Tgi) was utilized as a surrogate of core temperature and measured by a pre-calibrated ingestible temperature sensor (e-Celsius; Bodycap; Caen, France) consumed with food approximately one-to-three hours prior to work shift. The sensor measured and recorded Tgi every 30 seconds for download post-shift via a wireless hand-held receiver (e-Viewer; Bodycap; Caen, France). The mean of Tgi was calculated for five-minute periods.

Thermal sensation for the first-half and second-half of shift were assessed via the modified numeric and descriptive scales pioneered by Adolf Pharo Gagge and colleagues in 1967.^8^

### Fluid Balance

Urine specific gravity (USG) was assessed with a calibrated refractometer (Atago UG-a; Tokyo, Japan) as an indication of hydration status pre-shift. Prior to, and following the work shift, participants were weighed semi-nude on a portable calibrated platform scale (UC321 A&D Mercury; Adelaide, South Australia), accurate to 0.05kg. The resultant change in body mass was expressed as a percentage of pre-exercise body mass. There was easy access to cold fluids in the Green Zone. Fluid consumption was not possible whilst in PPE in Orange or Red Zones. Fluid consumption was self-monitored and urine volume was self-estimated. Dehydration and sweat loss were calculated as described by Matt Brearley and colleagues.^9^ Dehydration was expressed as a percentage of body mass by the following equation:

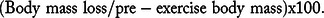




Sweat Loss (L) was calculated by the following equation:

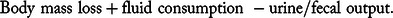




### Environmental Conditions

Ambient temperature, relative humidity, apparent temperature, wind speed, and outdoor Wet Bulb Globe Temperature (WBGT) were acquired from the Bureau of Meteorology Darwin Airport Weather Station (station number 014015; Darwin International Airport, Eaton NT, Australia) every 60 minutes during the period from 08:00am to 5:00pm across both monitoring days. The weather station is located 16.0km from the test site. The Apparent Temperature is defined as the temperature, at the reference humidity level, producing the same amount of discomfort as that experienced under the current ambient temperature and humidity. Apparent Temperature is also known as the Heat Index is some jurisdictions. While solar radiation was not accounted for by apparent temperature reported in this study, it was accounted for by the WBGT^10^ where black globe temperature was weighted at 20%, in conjunction with wet bulb temperature (70%) and ambient temperature (10%). The WBGT was developed explicitly to mitigate the risk of heat illness during physical exertion.

### Statistics

As an observational study, descriptive statistics are reported including mean and standard deviation (SD).

## Results

### Physiological Monitoring Study: Participant Characteristics

There were 18 participants (eight males and ten females) in the physiological component of the study. The mean age of the group was 41.4 years (SD = 11.5), mean height 1.69m (SD = 0.09), mean body mass 80.6kg (SD = 23.0), and mean years living in the Darwin region 9.0 years (SD = 5.7). Participants were classified into the following work groups: health (n = 8), welfare (n = 4), facilities/services (n = 4), and security/police (n = 2). Physiological data collected from each participant are described in Table 1.

**Table 1.** Physiological Data Collected on Each Participant

Participant	USG	Sweat Rate	Dehydration	Core Temperature (°C)	
		(L/hr)	(% Body Mass)	Peak	Mean
1	1.022	0.52	(0.4)	38.0	37.2
2	1.026	0.25	(0.2)	37.9	37.7
3	1.004	0.18	(0.5)	37.5	37.1
4	1.022	0.36	0.6	37.6	37.3
5	1.005	0.15	0.4	37.3	37.1
6	1.026	0.17	(1.6)	37.4	37.2
7	1.002	0.32	0.5	37.9	37.6
8	1.010	0.24	(1.0)	37.5	37.0
9	1.015	0.13	(1.2)	37.9	37.5
10	1.005	0.15	(0.4)	37.7	37.3
11	1.007	0.15	(0.6)	37.6	37.1
12	1.019	0.16	(1.1)	38.1	37.5
13	1.021	–	–	38.0	37.5
14	1.027	0.23	(0.5)	38.1	37.5
15	1.025	0.46	1.4	37.8	37.4
16	1.005	0.22	(0.6)	37.6	37.4
17	1.017	0.16	0.0	37.7	37.1
18	1.020	0.17	(0.5)	37.8	37.3

Abbreviation: USG, urine specific gravity.

### Environmental Conditions

Environmental conditions were measured in the first-half and second-half of the shift. The conditions were similar between shift segments, considered hot and humid and typical of the transitional period between dry and wet seasons (termed “build up”) in Northern Australia. The mean ambient temperature was 31.3°C (SD = 1.7) in the first-half of the day and 32.1°C (SD = 1.0) in the second-half of the day. The mean relative humidity was 63.8% (SD = 10.9) and 62.9% (SD = 5.1). The mean apparent temperature was 34.7°C (SD = 0.8) and 35.6°C (SD = 1.9). The mean WGBT was 33.1°C (SD = 0.6) and 34.0°C (SD = 0.3).

### Physiological Core Temperature

**Figure 1.**
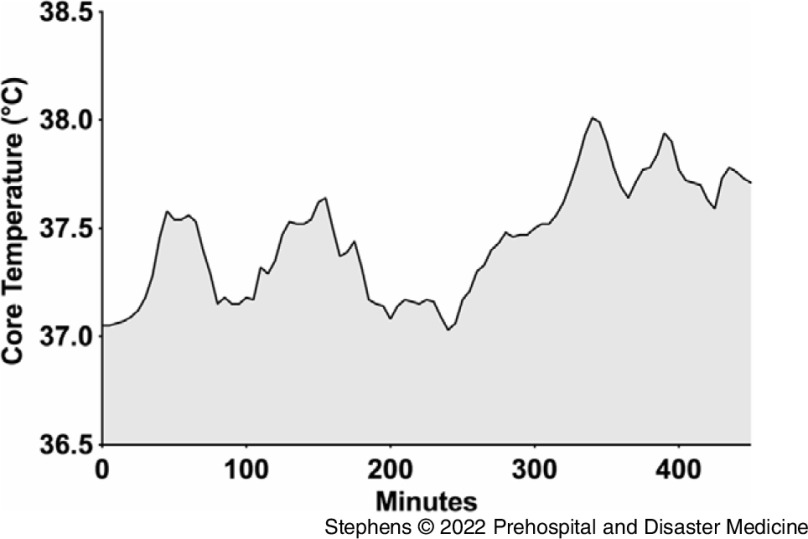
Fluctuations of Core Temperature Recorded in One Individual Over the Course of Their Shift.

The work shift duration mean was 10.1 (SD = 0.8) hours, and across the shift, the mean core temperature was 37.3°C (SD = 0.2) with a range of 37.0°C - 37.7°C. The mean of maximal temperature was 37.7°C (SD = 0.3) ranging from 37.5°C - 38.1°C. Figure 1 is an example of the fluctuations that occurred in one worker over the shift.

### Fluid Balance

The mean USG was 1.015 (SD = 0.009). The mean fluid consumption was 2.9L (SD = 1.1), mean urine volume 2.4L (SD = 1.1), and mean hourly sweat rate 250ml (SD = 110ml). The mean body mass increased by 0.3% (SD = 0.3) over the course of the shift.

### Subjective Heat Health Survey

The cumulative responses to the heat health survey are displayed in Table 2. The workforce is separated into those wearing PPE (entering Orange and Red Zones) and those not wearing PPE (confined to working in the Green Zone). In the workforce in PPE, 57% reported feeling moderately, severely, or unbearably hot compared to 49% of those not in PPE. Moderate to severe sweating was reported in 70% of those working in PPE compared to 56% of those not wearing PPE. The level of fatigue was reported as moderate to severe in just over 25% of the workforce in both groups.

**Table 2.** Subjective Responses of Workers Classified on the Basis of PPE

Variable	Descriptor	PPE (% of Respondents)	Non-PPE (% of Respondents)
Thermal Sensation	Not Hot at All	4.6	12.8
	Mildly Hot	37.9	38.5
	Moderately Hot	39.3	20.5
	Severely Hot	16.7	28.2
	Unbearably Hot	1.5	0.0
Level of Sweating	Minimal	30.3	43.6
	Moderate	43.9	33.3
	A Lot	19.7	18.0
	Severe	6.1	5.1
Level of Fatigue	None	26.7	28.2
	Minimal	46.8	43.6
	Moderate	23.4	20.5
	Severe	3.1	7.7
	Debilitating	0.0	0.0
Environmental Conditions	Comfortable	46.0	31.6
	Warm but Tolerable	34.9	36.8
	Hot	15.9	21.1
	Too Hot	3.2	10.5

Abbreviation: PPE, personal protective equipment.

## Discussion

The impact of heat and humidity on the health and well-being of outdoor workers has been documented in different occupational groups, including medical and emergency responders.^11–15^ The PPE worn by frontline outdoor emergency COVID-19 response workers protects the workforce from acquiring and transmitting disease but increases their vulnerability to heat stress and associated sequelae.^4^ The vulnerability and therefore the risk of heat stress is amplified in the tropics where environmental heat and humidity are a constant backdrop to the work.

The study confirms the impact of the environment on the workers in HSQF. The survey demonstrated that more than one-half of the workforce is experiencing subjective moderate to unbearable levels of heat at work, the majority experience excessive sweating, and one-quarter of the workforce feels significant fatigue as a consequence of their environmental exposure. The physiological study participants all had elevated core temperature during their shift, despite adequate hydration, underscoring the separate and significant impact of heat stress separate to dehydration and the need to address this component of exposure to the environment with strategies in addition to drinking enough fluids. The maximum core temperature achieved by any participant in this study was 38.1°C and compares favorably with similar studies in that excessive temperatures were avoided.^9,16^ The ambient conditions were similar to other studies, and the reasonable conclusion is heat health strategies existing at the HSQF workforce prior to this study have a positive impact on people limiting their exposure and therefore the risk of getting excessively hot.

The pandemic response has required an iterative and adaptive response. Hotel quarantine is a core pillar of protecting the Australian community from transmission events from international arrivals, but it is not fit for purpose from an infection control perspective, and the advantages of an accommodation village like HSQF has become evident.^1^ Other similar facilities are currently under construction in Victoria and Queensland. The strategies to mitigate the risk of heat stress in the quarantine workforce are applicable across jurisdictions and international boundaries on a seasonal basis.

The measurement of risk ranges from simple survey techniques to complex monitoring of core temperature over time. The survey provides the information required to monitor the well-being of the workforce, has the advantage of being suitable to administer in low-resource settings, and is easily repeated. Survey results can guide the implementation of simple risk mitigation strategies without the need for complex resource and time investment. The advantage of more complex core temperature monitoring systems is the capacity to track workers objectively throughout a shift. The monitoring of core temperature provides quantitative data and potentially evidence of the impact of interventions to lower core temperature throughout the day – this information can be used to inform employers of the most effective risk mitigation strategies for that workforce.

In HSQF, the strategies to reduce heat stress need to be pragmatic as workers are employed in an emergency response framework and must deliver on assigned work in a timely manner to ensure the safety of the residents, the workforce, and the community. The care of quarantined residents and the infection prevention PPE required to be worn must meet national health and safety standards. There are a hierarchy of controls available to mitigate the risk associated with heat stress in the quarantine environment.

**Figure 2.**
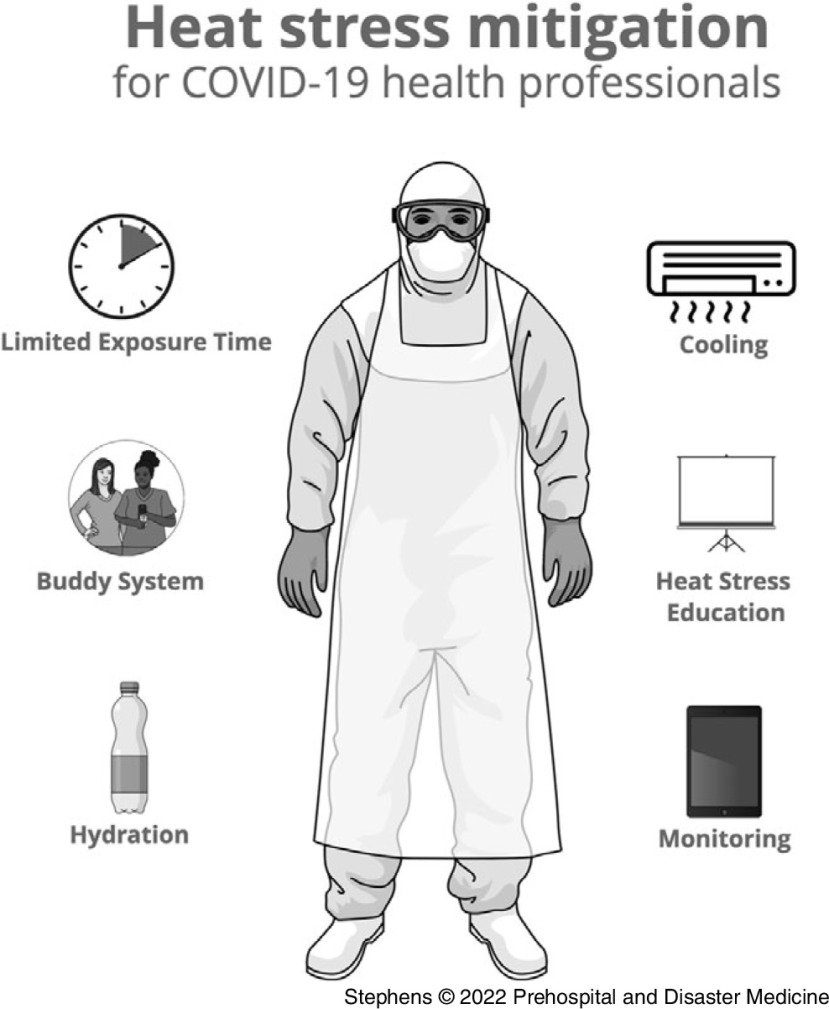
Infographic for Implementation and Promotion of Heat Health in the Workplace.

Heat stress impacts on the health and well-being of workers and increases the risk of workplace injury.^7^ In the quarantine setting, the additional risk of breaches in PPE and transmission events increases the stakes of heat stress with an outbreak potentially resulting in a regional lockdown with significant negative impact on the community and the economy. The HSQF Heat Health Working Group established the 10 steps to prevent heat-related illness (Table 3 and Figure 2). A complex mix of human factors may prevent workers from monitoring and responding to their own heat stress indicators. It is important in an emergency response environment that workers clearly understand the risks of heat stress and feel empowered and encouraged to “tap out” before symptoms of heat stress impact on their performance. Heat health should be included in all site inductions and reinforced with regular updates, posters, and clearly signed cool spaces. The site leadership team needs to demonstrate through their own actions the importance of taking heat health seriously. The impact of heat stress on cognitive function may result in PPE breaches. An important mitigation strategy to avoid PPE breaches in the heat employed at HSQF throughout the COVID-19 response is to have a strict buddy system. The buddy system is a strictly enforced rule requiring that every person going into the Red or Orange Zones must enter with a buddy who remains with them throughout the time in the zone. The buddy system ensures that every person donns PPE with a buddy, travels into the zone with a buddy, and exits the zone with a buddy. Buddies must closely observe each other donning and doffing to ensure it is done safely. Buddies are tasked with ensuring their workmate doesn’t get too hot and is empowered to suggest “tapping out” if one or both of them are showing early signs of heat-related illness.

**Table 3.** Howard Springs Quarantine Facility Heat Health Standard Operating Procedure

The 10 STEPS to PREVENTING HEAT-RELATED ILLNESS
Heat health training (site induction sessions/online presentation/WHS snapshot training). Avoid direct sun as much as possible. Plan your work load so that you are in the Orange Zone for the minimum time possible. Self-monitor and remove yourself from the heat for a cool break if you are feeling affected by heat – let your buddy and team leader know you need to have a cool break. Monitor your workmates and suggest a cool down break if they appear to be affected by the heat. If your task in the Orange Zone requires longer than one hour, you must have a cool break for at least 10 minutes every hour – set an alarm to remind you. Doff your PPE with your buddy and take your cool down break, can be in your pod or office space or in the staff dining area. Plan to ingest ice during cool down to lower core temperature more effectively - Slushy machines/Zooper Doopers (flavored ice sticks) are available to staff for this purpose. Ensure hydration – drink to your thirst level and based upon your experience. At end of shift, at home use air-conditioned environment, cool shower, pool, and/or ice slushies to accelerate your drop in core temperature and your recovery. If you are still sweating following your shower, you require additional cooling. Eat healthy meals and avoid skipping meals. **NT Health CNR Heat Health Standard Operating Procedure August 2021**

Abbreviations: PPE, personal protective equipment; NT, Northern Territory; CNR, Centre for National Resilience.

Whilst avoiding direct sun with shade structures and wearing of hats is important, it remains hot in the shade in the tropics. Workers are encouraged to drink cool water and remain hydrated to mitigate the effects of dehydration that can compound the effects of heat stress. There is a time limit to working safely in the heat of the tropics that will vary according to the workers level of acclimatization. The HSQF uses a limit of 50 minutes in the Red or Orange Zone and workers are encouraged to enter cool spaces for 10 minutes at least every hour. In order to provide this opportunity, air-conditioned accommodation rooms were converted to cool spaces in each zone so workers can doff, cool down with a cold drink, and then donn and return to their work without having to proceed to an exit to go to a distant Green Zone. Ice slurry in the form of slushies and flavored ice blocks are additional simple methods used to effectively lower workers’ core temperature during working hours^17^ – these are available at PPE stations during high-intensity activities such as large intakes.

Finally, the quality of PPE impacts on heat management with plastic gowns performing worse than gowns made from breathable fabric because plastic gowns have higher thermal resistance and lower water vapor permeability resulting in increased heat and sweat trapping.

## Limitations

The study examined a relatively small number of workers in the physiological study and the anonymous survey prevented tracking an individual’s survey results over time. The convenience sampling used for the survey introduces additional risk of sampling error and bias in the results.

## Conclusion

Heat stress is a significant risk in outdoor workers in the tropics and is amplified in the COVID-19 frontline workforce required to wear PPE in outdoor settings. The study demonstrated significant heat stress in the HSQF workforce, underscoring the importance of mitigating the risk associated with heat stress in this critical frontline workforce. A heat health program aimed at mitigating risk including workplace education, limiting exposure times, encouraging hydration, buddy system, active cooling, and monitoring is recommended to limit PPE breaches and other workplace injuries in this workforce.
